# Bis(*N*,*N*-diethyl­dithio­carbamato)(1,10-phenanthroline)cobalt(III) tetra­fluorido­borate

**DOI:** 10.1107/S160053680801725X

**Published:** 2008-06-13

**Authors:** Peter D. W. Boyd, Clifton E. F. Rickard

**Affiliations:** a Department of Chemistry, The University of Auckland, Private Bag 92019, Auckland, New Zealand

## Abstract

The cationic complex in the structure of the title compound, [Co(Et_2_NCS_2_)_2_(C_12_H_8_N_2_)]BF_4_, has a Co^III^ atom with a distorted octa­hedral coordination formed by four S atoms of two diethyl­dithio­carbamate and two N atoms of 1,10-phenanthroline ligands. The crystal structure features head-to-tail stacking of the phenanthroline ligands. The tetra­fluorido­borate anions are positioned in the channels between the cation stacks running along the *a* axis, and form weak C—H⋯F interactions.

## Related literature

For other bis­(dialkyl­dithio­carbamato)*L*_2_cobalt(III) complexes (*L*_2_ = bis­monodentate or bidentate ligands), see: Bhardwaj & Aftab (1990 ▶); Deplano & Trogu (1982 ▶); Deplano *et al.* (1983 ▶); Hendrickson *et al.* (1975 ▶); Holah & Murphy (1971 ▶); McCleverty *et al.* (1977 ▶); Okuno *et al.* (1989 ▶); Hodgson *et al.* (2008 ▶); Ware *et al.* (1998 ▶).
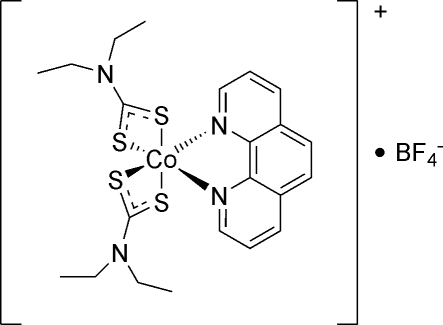


## Experimental

### 

#### Crystal data

[Co(C_5_H_10_NS_2_)_2_(C_12_H_8_N_2_)]BF_4_
*M* *_r_* = 622.46
Monoclinic, 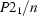

*a* = 8.0064 (1) Å
*b* = 16.3421 (3) Å
*c* = 21.0927 (3) Å
β = 95.013 (1)°
*V* = 2749.24 (7) Å^3^
*Z* = 4
Mo *K*α radiation
μ = 0.97 mm^−1^
*T* = 203 (2) K
0.38 × 0.12 × 0.06 mm

#### Data collection

Siemens SMART CCD diffractometer
Absorption correction: multi-scan (*SADABS*; Sheldrick, 1996 ▶) *T*_min_ = 0.761, *T*_max_ = 0.944
16341 measured reflections
5982 independent reflections
4410 reflections with *I* > 2σ(*I*)
*R*_int_ = 0.025

#### Refinement

*R*[*F* ^2^ > 2σ(*F* ^2^)] = 0.046
*wR*(*F* ^2^) = 0.099
*S* = 1.07
5982 reflections
325 parameters
24 restraints
H-atom parameters constrained
Δρ_max_ = 0.49 e Å^−3^
Δρ_min_ = −0.44 e Å^−3^

### 

Data collection: *SMART* (Siemens, 1995 ▶); cell refinement: *SAINT* (Siemens, 1995 ▶); data reduction: *SAINT*; program(s) used to solve structure: *SHELXS97* (Sheldrick, 2008 ▶); program(s) used to refine structure: *SHELXL97* (Sheldrick, 2008 ▶); molecular graphics: *Mercury* (Macrae *et al.*, 2006 ▶); software used to prepare material for publication: *WinGX* (Farrugia, 1999 ▶).

## Supplementary Material

Crystal structure: contains datablocks global, I. DOI: 10.1107/S160053680801725X/ya2076sup1.cif

Structure factors: contains datablocks I. DOI: 10.1107/S160053680801725X/ya2076Isup2.hkl

Additional supplementary materials:  crystallographic information; 3D view; checkCIF report

## Figures and Tables

**Table:** 

Co—S1	2.2805 (8)
Co—S2	2.2432 (8)
Co—S3	2.2590 (9)
Co—S4	2.2658 (8)
Co—N1	1.989 (2)
Co—N2	1.991 (2)

**Table:** 

N1—Co—N2	82.86 (9)
N1—Co—S2	171.16 (7)
N2—Co—S2	94.35 (7)
N1—Co—S3	93.48 (7)
N2—Co—S3	165.93 (7)
S2—Co—S3	91.18 (3)
N1—Co—S4	93.98 (7)
N2—Co—S4	89.75 (7)
S2—Co—S4	94.39 (3)
S3—Co—S4	76.91 (3)
N1—Co—S1	95.48 (7)
N2—Co—S1	97.99 (7)
S2—Co—S1	76.56 (3)
S3—Co—S1	95.87 (3)
S4—Co—S1	168.45 (3)

**Table 2:** C-H⋯F contacts (Å, °)

D—H	A	D—H	H⋯A	D⋯A	D—H.·A
C10—H10*A*	F1^i^	0.94	2.31	3.169 (4)	151
C2—H2*A*	F2^ii^	0.94	2.43	3.281 (4)	151
C6—H6*A*	F4^iv^	0.94	2.44	3.053 (4)	123

Symmetry codes (i) 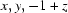; (ii) 
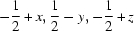
; (iii) 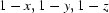; (iv) 
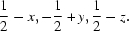

**Fig. 1.**
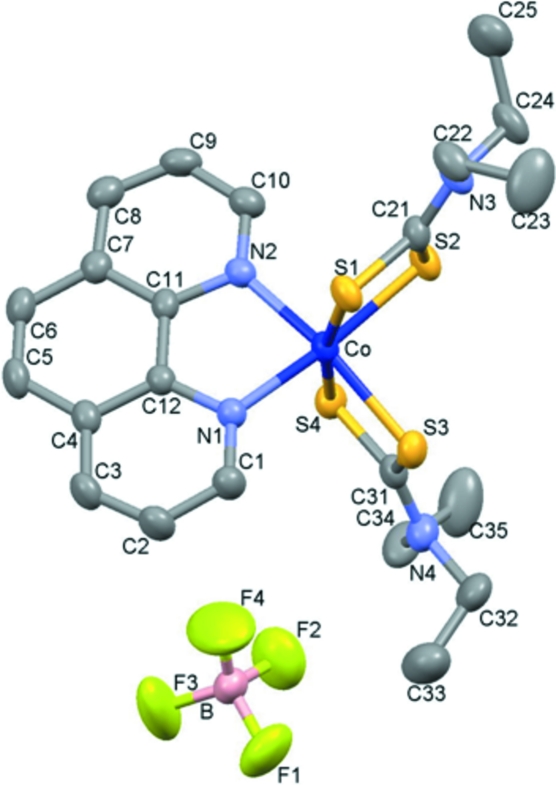
Structure of (I) showing 50% probability displacement ellipsoids; the H atoms are omitted for clarity.

**Fig. 2.**
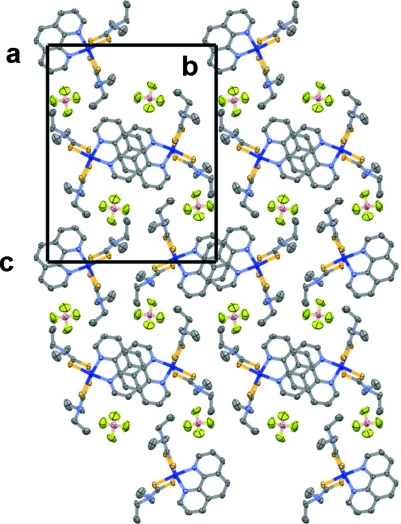
The crystal packing of the title compound viewed along the a axis showing stacking of phenanthroline ligands as well as the channels between cation stacks occupied by the BF4- ions.

## supplementary crystallographic information

### Comment

The reaction of the bimetallic cobalt(III) complex [Co_2_(Et_2_NCS_2_)_5_]BF_4_ (Hendrickson *et al.*, 1975) with bidentate neutral and anionic ligands (*L*) has provided a convenient route for the preparation of mixed ligand cobalt(III) bisdithiocarbamate complexes such as [Co(Et_2_NCS_2_)_2_L]BF_4_ (McCleverty *et al., *1977; Deplano & Trogu, 1982; Deplano *et al.*, 1983; Ware *et al., *1998; Hodgson *et al.*,  2008). Several papers on the preparation of diimine complexes with *L*=2,2'-bipyridine or 1,10-phenantholine have been published (Holah & Murphy, 1971; Okuno *et al., *1989; Bhardwaj & Aftab, 1990; Hodgson *et al.*, 2008). In the present communication the crystal structure of [Co(Et_2_NCS_2_)_2_L]BF_4_ (*L*=1,10-phenanthroline)(I), formed by reaction of [Co_2_((C_2_H_5_)_2_NCS_2_)_5_]BF_4_ with 1,10-phenanthroline, is reported.

The molecular structure of (I) is shown in Fig. 1. The Co atom has a distorted octahedral coordination formed by four S atoms of two dithiocarbamate and two N atoms of 1,10-phenanthroline ligands (Table 1).

The crystal packing of the title compound (Fig. 2) features head-to-tail cationic complexes assembled in the crystal *via* stacking of the phenanthroline ligands in an alternating mode (interplanar distance is 3.57 Å). The tetrafluoroborate anions are located in the channels between the cation stacks running along the *a* axis of the structure and are held in position by many C—H···F interactions between phenanthroline C—H bonds and the F atoms of the tetrafluoroborate anion, (Table 2).

### Experimental

The complex(I) was prepared by reaction of equimolar amounts of [Co_2_((C_2_H_5_)_2_NCS_2_)_5_]BF_4 _ (Hendrickson *et al.*, 1975) and 1,10-phenanthroline in dichloromethane solution at room temperature following the same procedure to that reported for the synthesis of the analogous dimethyldithiocarbamate complex (Hodgson *et al.*, 2008). Crystals were grown from a dichloromethane solution.

### Refinement

Hydrogen atoms were placed in calculated positions and refined using the riding model [C—H 0.93–0.97 Å), with *U*_iso_(H) = 1.2 *U*_eq_(C) for aromatic and methylene groups  and 1.5*U*_eq_(C) for methyl groups. In the case of the methyl groups, protons were rotated to fit the H-atom positions to the observed electron density. SHELXL97 retraints SIMU and DELU (Sheldrick, 2008) were applied to the thermal parameters for the fluorine atoms of the tetrafluoroborate anions.

### Crystal data

**Table:**

[Co(C_5_H_10_NS_2_)_2_(C_12_H_8_N_2_)]BF_4_	*F*_000_ = 1280
*M**_r_* = 622.46	*D*_x_ = 1.504 Mg m^−^^3^
Monoclinic, *P*2_1_/*n*	Mo *K*α radiation λ = 0.71073 Å
*a* = 8.0064 (1) Å	Cell parameters from 8163 reflections
*b* = 16.3421 (3) Å	θ = 1.9–27.5º
*c* = 21.0927 (3) Å	µ = 0.97 mm^−^^1^
β = 95.013 (1)º	*T* = 203 (2) K
*V* = 2749.24 (7) Å^3^	Needle, red
*Z* = 4	0.38 × 0.12 × 0.06 mm

### Data collection

**Table:**

Siemens SMART CCD diffractometer	5982 independent reflections
Radiation source: fine-focus sealed tube	4410 reflections with *I* > 2σ(*I*)
Monochromator: graphite	*R*_int_ = 0.025
*T* = 203(2) K	θ_max_ = 27.5º
ω scans	θ_min_ = 1.9º
Absorption correction: multi-scan(SADABS; Sheldrick, 1996)	*h* = −10→10
*T*_min_ = 0.761, *T*_max_ = 0.944	*k* = −19→20
16341 measured reflections	*l* = −17→27

### Refinement

**Table:**

Refinement on *F*^2^	Secondary atom site location: difference Fourier map
Least-squares matrix: full	Hydrogen site location: inferred from neighbouring sites
*R*[*F*^2^ > 2σ(*F*^2^)] = 0.046	H-atom parameters constrained
*wR*(*F*^2^) = 0.099	*w* = 1/[σ^2^(*F*_o_^2^) + (0.023*P*)^2^ + 3.5289*P*] where *P* = (*F*_o_^2^ + 2*F*_c_^2^)/3
*S* = 1.07	(Δ/σ)_max_ < 0.001
5982 reflections	Δρ_max_ = 0.49 e Å^−^^3^
325 parameters	Δρ_min_ = −0.44 e Å^−^^3^
24 restraints	Extinction correction: none
Primary atom site location: structure-invariant direct methods	

### Special details

**Table:**

Geometry. All esds (except the esd in the dihedral angle between two l.s. planes) are estimated using the full covariance matrix. The cell esds are taken into account individually in the estimation of esds in distances, angles and torsion angles; correlations between esds in cell parameters are only used when they are defined by crystal symmetry. An approximate (isotropic) treatment of cell esds is used for estimating esds involving l.s. planes.
Refinement. Refinement of F^2^ against ALL reflections. The weighted R-factor wR and goodness of fit S are based on F^2^, conventional R-factors R are based on F, with F set to zero for negative F^2^. The threshold expression of F^2^ > 2sigma(F^2^) is used only for calculating R-factors(gt) etc. and is not relevant to the choice of reflections for refinement. R-factors based on F^2^ are statistically about twice as large as those based on F, and R- factors based on ALL data will be even larger.

### Fractional atomic coordinates and isotropic or equivalent isotropic displacement parameters (Å^2^)

**Table:**

	*x*	*y*	*z*	*U*_iso_*/*U*_eq_	
Co	0.18850 (5)	0.24157 (2)	0.017869 (19)	0.02837 (11)	
S1	−0.08717 (9)	0.25666 (4)	−0.01750 (4)	0.03400 (18)	
S2	0.18002 (9)	0.36489 (4)	−0.02902 (4)	0.03424 (18)	
S3	0.16723 (10)	0.29916 (5)	0.11419 (4)	0.03561 (19)	
S4	0.45935 (9)	0.25371 (5)	0.05787 (4)	0.03378 (18)	
N1	0.1650 (3)	0.12827 (14)	0.05041 (11)	0.0287 (5)	
N2	0.2630 (3)	0.18340 (14)	−0.05766 (11)	0.0296 (5)	
N3	−0.1280 (3)	0.39591 (15)	−0.08739 (13)	0.0369 (6)	
N4	0.4748 (3)	0.31052 (16)	0.17814 (13)	0.0405 (6)	
C1	0.1077 (4)	0.10206 (19)	0.10422 (15)	0.0360 (7)	
H1A	0.0660	0.1406	0.1319	0.043*	
C2	0.1072 (4)	0.01878 (19)	0.12111 (16)	0.0405 (8)	
H2A	0.0649	0.0024	0.1593	0.049*	
C3	0.1684 (4)	−0.03832 (18)	0.08182 (16)	0.0372 (7)	
H3A	0.1702	−0.0939	0.0932	0.045*	
C4	0.2289 (4)	−0.01328 (17)	0.02424 (15)	0.0310 (6)	
C5	0.2980 (4)	−0.06714 (17)	−0.02050 (16)	0.0359 (7)	
H5A	0.3055	−0.1234	−0.0115	0.043*	
C6	0.3526 (4)	−0.03887 (18)	−0.07522 (16)	0.0373 (7)	
H6A	0.3979	−0.0758	−0.1033	0.045*	
C7	0.3427 (4)	0.04682 (18)	−0.09137 (14)	0.0319 (7)	
C8	0.3982 (4)	0.0808 (2)	−0.14704 (15)	0.0397 (8)	
H8A	0.4425	0.0472	−0.1776	0.048*	
C9	0.3868 (4)	0.1636 (2)	−0.15598 (15)	0.0410 (8)	
H9A	0.4256	0.1872	−0.1926	0.049*	
C10	0.3178 (4)	0.21330 (19)	−0.11098 (15)	0.0368 (7)	
H10A	0.3097	0.2699	−0.1186	0.044*	
C11	0.2767 (3)	0.10037 (16)	−0.04794 (14)	0.0277 (6)	
C12	0.2215 (3)	0.07098 (16)	0.01001 (13)	0.0273 (6)	
C21	−0.0290 (4)	0.34776 (17)	−0.05069 (15)	0.0312 (7)	
C22	−0.3106 (4)	0.38155 (19)	−0.09710 (17)	0.0419 (8)	
H22A	−0.3370	0.3281	−0.0793	0.050*	
H22B	−0.3455	0.3806	−0.1428	0.050*	
C23	−0.4057 (5)	0.4470 (3)	−0.0658 (3)	0.0801 (15)	
H23A	−0.5250	0.4362	−0.0729	0.120*	
H23B	−0.3810	0.4998	−0.0839	0.120*	
H23C	−0.3726	0.4474	−0.0205	0.120*	
C24	−0.0615 (4)	0.46964 (19)	−0.11725 (17)	0.0438 (8)	
H24A	0.0600	0.4722	−0.1068	0.053*	
H24B	−0.1111	0.5184	−0.0994	0.053*	
C25	−0.0982 (5)	0.4703 (2)	−0.18846 (19)	0.0620 (11)	
H25A	−0.0524	0.5197	−0.2057	0.093*	
H25B	−0.2185	0.4689	−0.1991	0.093*	
H25C	−0.0472	0.4228	−0.2065	0.093*	
C31	0.3820 (4)	0.29130 (17)	0.12572 (15)	0.0329 (7)	
C32	0.4009 (5)	0.3398 (2)	0.23524 (16)	0.0501 (9)	
H32A	0.2940	0.3670	0.2226	0.060*	
H32B	0.4759	0.3803	0.2570	0.060*	
C33	0.3713 (6)	0.2702 (3)	0.2810 (2)	0.0727 (13)	
H33A	0.3222	0.2919	0.3180	0.109*	
H33B	0.4772	0.2440	0.2944	0.109*	
H33C	0.2956	0.2305	0.2599	0.109*	
C34	0.6587 (4)	0.3015 (2)	0.18175 (19)	0.0574 (10)	
H34A	0.6872	0.2552	0.1552	0.069*	
H34B	0.7009	0.2894	0.2258	0.069*	
C35	0.7437 (5)	0.3778 (3)	0.1599 (3)	0.0833 (16)	
H35A	0.8640	0.3694	0.1630	0.125*	
H35B	0.7176	0.4236	0.1866	0.125*	
H35C	0.7039	0.3894	0.1160	0.125*	
B	0.8580 (6)	0.1060 (2)	0.25563 (19)	0.0456 (10)	
F1	0.7989 (3)	0.11912 (17)	0.31314 (12)	0.0873 (8)	
F2	0.9614 (4)	0.16960 (17)	0.24311 (15)	0.0952 (9)	
F3	0.9452 (5)	0.03466 (17)	0.25818 (16)	0.1194 (13)	
F4	0.7309 (5)	0.1022 (2)	0.21044 (15)	0.1376 (14)	

### Atomic displacement parameters (Å^2^)

**Table:**

	*U*^11^	*U*^22^	*U*^33^	*U*^12^	*U*^13^	*U*^23^
Co	0.0281 (2)	0.02278 (19)	0.0343 (2)	0.00121 (16)	0.00333 (16)	0.00146 (17)
S1	0.0298 (4)	0.0249 (4)	0.0470 (5)	−0.0036 (3)	0.0017 (3)	0.0067 (3)
S2	0.0272 (4)	0.0250 (4)	0.0502 (5)	−0.0023 (3)	0.0017 (3)	0.0057 (3)
S3	0.0340 (4)	0.0307 (4)	0.0428 (5)	0.0030 (3)	0.0074 (3)	−0.0053 (3)
S4	0.0285 (4)	0.0332 (4)	0.0396 (4)	0.0043 (3)	0.0032 (3)	−0.0035 (3)
N1	0.0299 (13)	0.0264 (12)	0.0297 (13)	−0.0001 (10)	0.0025 (10)	0.0002 (10)
N2	0.0306 (13)	0.0284 (13)	0.0294 (13)	0.0003 (10)	0.0001 (10)	0.0027 (10)
N3	0.0284 (13)	0.0289 (13)	0.0526 (17)	−0.0021 (10)	−0.0012 (12)	0.0097 (12)
N4	0.0423 (16)	0.0350 (14)	0.0433 (16)	0.0025 (12)	−0.0014 (13)	−0.0070 (12)
C1	0.0387 (17)	0.0347 (16)	0.0355 (17)	0.0014 (13)	0.0086 (14)	0.0015 (14)
C2	0.0456 (19)	0.0361 (17)	0.0407 (19)	−0.0025 (14)	0.0093 (15)	0.0087 (15)
C3	0.0386 (18)	0.0262 (15)	0.0469 (19)	−0.0030 (13)	0.0045 (15)	0.0074 (14)
C4	0.0278 (15)	0.0261 (14)	0.0384 (17)	−0.0004 (11)	−0.0014 (13)	−0.0004 (13)
C5	0.0340 (17)	0.0218 (14)	0.052 (2)	−0.0013 (12)	0.0013 (14)	−0.0027 (14)
C6	0.0373 (17)	0.0304 (16)	0.0441 (19)	0.0002 (13)	0.0030 (14)	−0.0093 (14)
C7	0.0290 (16)	0.0328 (15)	0.0333 (17)	−0.0006 (12)	−0.0001 (13)	−0.0030 (13)
C8	0.0390 (18)	0.0471 (19)	0.0328 (18)	0.0015 (15)	0.0021 (14)	−0.0047 (15)
C9	0.0430 (19)	0.053 (2)	0.0271 (16)	−0.0004 (15)	0.0023 (14)	0.0070 (15)
C10	0.0385 (18)	0.0377 (17)	0.0339 (17)	0.0004 (14)	0.0006 (14)	0.0069 (14)
C11	0.0252 (14)	0.0246 (14)	0.0324 (16)	0.0008 (11)	−0.0024 (12)	−0.0036 (12)
C12	0.0258 (14)	0.0265 (14)	0.0293 (16)	0.0000 (11)	0.0002 (12)	−0.0003 (12)
C21	0.0307 (16)	0.0233 (14)	0.0397 (17)	−0.0003 (11)	0.0039 (13)	−0.0019 (13)
C22	0.0291 (16)	0.0364 (17)	0.059 (2)	−0.0033 (13)	−0.0038 (15)	0.0119 (16)
C23	0.036 (2)	0.089 (3)	0.118 (4)	−0.004 (2)	0.018 (2)	−0.035 (3)
C24	0.0381 (18)	0.0285 (16)	0.064 (2)	−0.0036 (13)	0.0017 (16)	0.0164 (16)
C25	0.074 (3)	0.052 (2)	0.062 (3)	−0.007 (2)	0.015 (2)	0.013 (2)
C31	0.0360 (17)	0.0242 (14)	0.0381 (17)	0.0012 (12)	0.0016 (14)	0.0002 (13)
C32	0.065 (2)	0.045 (2)	0.040 (2)	−0.0035 (17)	0.0006 (17)	−0.0140 (16)
C33	0.098 (4)	0.071 (3)	0.049 (2)	−0.013 (3)	0.009 (2)	−0.002 (2)
C34	0.045 (2)	0.063 (2)	0.060 (2)	0.0132 (18)	−0.0192 (18)	−0.022 (2)
C35	0.043 (2)	0.075 (3)	0.134 (5)	−0.010 (2)	0.018 (3)	−0.037 (3)
B	0.058 (3)	0.041 (2)	0.037 (2)	0.0036 (19)	0.0057 (19)	−0.0055 (18)
F1	0.100 (2)	0.102 (2)	0.0643 (16)	−0.0059 (16)	0.0286 (15)	−0.0340 (15)
F2	0.091 (2)	0.0754 (18)	0.123 (2)	−0.0163 (15)	0.0321 (18)	0.0104 (17)
F3	0.179 (3)	0.0618 (17)	0.129 (3)	0.0507 (19)	0.080 (3)	0.0205 (17)
F4	0.178 (3)	0.130 (3)	0.090 (2)	−0.036 (3)	−0.070 (2)	−0.003 (2)

### Geometric parameters (Å, °)

**Table:**

Co—S1	2.2805 (8)	C8—C9	1.367 (5)
Co—S2	2.2432 (8)	C8—H8A	0.9400
Co—S3	2.2590 (9)	C9—C10	1.400 (4)
Co—S4	2.2658 (8)	C9—H9A	0.9400
Co—N1	1.989 (2)	C10—H10A	0.9400
Co—N2	1.991 (2)	C11—C12	1.419 (4)
S1—C21	1.726 (3)	C22—C23	1.499 (5)
S2—C21	1.719 (3)	C22—H22A	0.9800
S3—C31	1.720 (3)	C22—H22B	0.9800
S4—C31	1.722 (3)	C23—H23A	0.9700
N1—C1	1.332 (4)	C23—H23B	0.9700
N1—C12	1.369 (3)	C23—H23C	0.9700
N2—C10	1.335 (4)	C24—C25	1.505 (5)
N2—C11	1.375 (3)	C24—H24A	0.9800
N3—C21	1.319 (4)	C24—H24B	0.9800
N3—C22	1.477 (4)	C25—H25A	0.9700
N3—C24	1.480 (4)	C25—H25B	0.9700
N4—C31	1.315 (4)	C25—H25C	0.9700
N4—C32	1.468 (4)	C32—C33	1.524 (5)
N4—C34	1.475 (4)	C32—H32A	0.9800
C1—C2	1.407 (4)	C32—H32B	0.9800
C1—H1A	0.9400	C33—H33A	0.9700
C2—C3	1.367 (4)	C33—H33B	0.9700
C2—H2A	0.9400	C33—H33C	0.9700
C3—C4	1.407 (4)	C34—C35	1.512 (6)
C3—H3A	0.9400	C34—H34A	0.9800
C4—C12	1.409 (4)	C34—H34B	0.9800
C4—C5	1.437 (4)	C35—H35A	0.9700
C5—C6	1.351 (4)	C35—H35B	0.9700
C5—H5A	0.9400	C35—H35C	0.9700
C6—C7	1.442 (4)	B—F4	1.333 (5)
C6—H6A	0.9400	B—F3	1.357 (5)
C7—C11	1.403 (4)	B—F1	1.357 (5)
C7—C8	1.406 (4)	B—F2	1.369 (5)
			
N1—Co—N2	82.86 (9)	N1—C12—C4	123.1 (3)
N1—Co—S2	171.16 (7)	N1—C12—C11	116.7 (2)
N2—Co—S2	94.35 (7)	C4—C12—C11	120.2 (3)
N1—Co—S3	93.48 (7)	N3—C21—S2	125.5 (2)
N2—Co—S3	165.93 (7)	N3—C21—S1	125.6 (2)
S2—Co—S3	91.18 (3)	S2—C21—S1	108.88 (16)
N1—Co—S4	93.98 (7)	N3—C22—C23	111.1 (3)
N2—Co—S4	89.75 (7)	N3—C22—H22A	109.4
S2—Co—S4	94.39 (3)	C23—C22—H22A	109.4
S3—Co—S4	76.91 (3)	N3—C22—H22B	109.4
N1—Co—S1	95.48 (7)	C23—C22—H22B	109.4
N2—Co—S1	97.99 (7)	H22A—C22—H22B	108.0
S2—Co—S1	76.56 (3)	C22—C23—H23A	109.5
S3—Co—S1	95.87 (3)	C22—C23—H23B	109.5
S4—Co—S1	168.45 (3)	H23A—C23—H23B	109.5
C21—S1—Co	86.45 (10)	C22—C23—H23C	109.5
C21—S2—Co	87.84 (10)	H23A—C23—H23C	109.5
C31—S3—Co	86.73 (11)	H23B—C23—H23C	109.5
C31—S4—Co	86.48 (10)	N3—C24—C25	112.6 (3)
C1—N1—C12	117.9 (2)	N3—C24—H24A	109.1
C1—N1—Co	130.1 (2)	C25—C24—H24A	109.1
C12—N1—Co	112.05 (18)	N3—C24—H24B	109.1
C10—N2—C11	117.3 (3)	C25—C24—H24B	109.1
C10—N2—Co	130.0 (2)	H24A—C24—H24B	107.8
C11—N2—Co	112.17 (19)	C24—C25—H25A	109.5
C21—N3—C22	121.6 (2)	C24—C25—H25B	109.5
C21—N3—C24	121.0 (2)	H25A—C25—H25B	109.5
C22—N3—C24	117.3 (2)	C24—C25—H25C	109.5
C31—N4—C32	121.9 (3)	H25A—C25—H25C	109.5
C31—N4—C34	120.5 (3)	H25B—C25—H25C	109.5
C32—N4—C34	117.6 (3)	N4—C31—S3	125.8 (2)
N1—C1—C2	122.4 (3)	N4—C31—S4	124.5 (2)
N1—C1—H1A	118.8	S3—C31—S4	109.69 (17)
C2—C1—H1A	118.8	N4—C32—C33	111.9 (3)
C3—C2—C1	119.9 (3)	N4—C32—H32A	109.2
C3—C2—H2A	120.1	C33—C32—H32A	109.2
C1—C2—H2A	120.1	N4—C32—H32B	109.2
C2—C3—C4	119.6 (3)	C33—C32—H32B	109.2
C2—C3—H3A	120.2	H32A—C32—H32B	107.9
C4—C3—H3A	120.2	C32—C33—H33A	109.5
C3—C4—C12	117.2 (3)	C32—C33—H33B	109.5
C3—C4—C5	124.8 (3)	H33A—C33—H33B	109.5
C12—C4—C5	118.1 (3)	C32—C33—H33C	109.5
C6—C5—C4	121.6 (3)	H33A—C33—H33C	109.5
C6—C5—H5A	119.2	H33B—C33—H33C	109.5
C4—C5—H5A	119.2	N4—C34—C35	112.0 (3)
C5—C6—C7	121.3 (3)	N4—C34—H34A	109.2
C5—C6—H6A	119.4	C35—C34—H34A	109.2
C7—C6—H6A	119.4	N4—C34—H34B	109.2
C11—C7—C8	117.6 (3)	C35—C34—H34B	109.2
C11—C7—C6	117.9 (3)	H34A—C34—H34B	107.9
C8—C7—C6	124.5 (3)	C34—C35—H35A	109.5
C9—C8—C7	118.9 (3)	C34—C35—H35B	109.5
C9—C8—H8A	120.5	H35A—C35—H35B	109.5
C7—C8—H8A	120.5	C34—C35—H35C	109.5
C8—C9—C10	120.4 (3)	H35A—C35—H35C	109.5
C8—C9—H9A	119.8	H35B—C35—H35C	109.5
C10—C9—H9A	119.8	F4—B—F3	110.3 (4)
N2—C10—C9	122.5 (3)	F4—B—F1	110.0 (4)
N2—C10—H10A	118.8	F3—B—F1	108.6 (3)
C9—C10—H10A	118.8	F4—B—F2	109.5 (4)
N2—C11—C7	123.2 (3)	F3—B—F2	110.0 (4)
N2—C11—C12	115.8 (2)	F1—B—F2	108.4 (3)
C7—C11—C12	121.0 (3)		
			
N1—Co—S1—C21	−172.69 (12)	C6—C7—C8—C9	−177.9 (3)
N2—Co—S1—C21	−89.16 (12)	C7—C8—C9—C10	−1.3 (5)
S2—Co—S1—C21	3.43 (10)	C11—N2—C10—C9	0.0 (4)
S3—Co—S1—C21	93.23 (10)	Co—N2—C10—C9	171.2 (2)
S4—Co—S1—C21	42.5 (2)	C8—C9—C10—N2	1.0 (5)
N2—Co—S2—C21	93.75 (12)	C10—N2—C11—C7	−0.8 (4)
S3—Co—S2—C21	−99.20 (10)	Co—N2—C11—C7	−173.5 (2)
S4—Co—S2—C21	−176.16 (10)	C10—N2—C11—C12	178.4 (2)
S1—Co—S2—C21	−3.44 (10)	Co—N2—C11—C12	5.7 (3)
N1—Co—S3—C31	90.50 (12)	C8—C7—C11—N2	0.6 (4)
N2—Co—S3—C31	16.2 (3)	C6—C7—C11—N2	179.1 (3)
S2—Co—S3—C31	−97.02 (10)	C8—C7—C11—C12	−178.6 (3)
S4—Co—S3—C31	−2.78 (10)	C6—C7—C11—C12	0.0 (4)
S1—Co—S3—C31	−173.62 (10)	C1—N1—C12—C4	−3.1 (4)
N1—Co—S4—C31	−89.87 (12)	Co—N1—C12—C4	175.6 (2)
N2—Co—S4—C31	−172.70 (12)	C1—N1—C12—C11	178.0 (3)
S2—Co—S4—C31	92.96 (10)	Co—N1—C12—C11	−3.3 (3)
S3—Co—S4—C31	2.78 (10)	C3—C4—C12—N1	2.5 (4)
S1—Co—S4—C31	55.0 (2)	C5—C4—C12—N1	−177.0 (3)
N2—Co—N1—C1	−176.5 (3)	C3—C4—C12—C11	−178.6 (3)
S3—Co—N1—C1	17.1 (3)	C5—C4—C12—C11	1.8 (4)
S4—Co—N1—C1	94.2 (3)	N2—C11—C12—N1	−1.6 (4)
S1—Co—N1—C1	−79.1 (3)	C7—C11—C12—N1	177.6 (2)
N2—Co—N1—C12	4.96 (19)	N2—C11—C12—C4	179.4 (3)
S3—Co—N1—C12	−161.39 (18)	C7—C11—C12—C4	−1.4 (4)
S4—Co—N1—C12	−84.29 (18)	C22—N3—C21—S2	−172.4 (2)
S1—Co—N1—C12	102.36 (18)	C24—N3—C21—S2	3.7 (4)
N1—Co—N2—C10	−177.3 (3)	C22—N3—C21—S1	8.5 (4)
S2—Co—N2—C10	11.1 (3)	C24—N3—C21—S1	−175.3 (2)
S3—Co—N2—C10	−101.7 (4)	Co—S2—C21—N3	−174.5 (3)
S4—Co—N2—C10	−83.3 (3)	Co—S2—C21—S1	4.67 (14)
S1—Co—N2—C10	88.1 (3)	Co—S1—C21—N3	174.6 (3)
N1—Co—N2—C11	−5.82 (19)	Co—S1—C21—S2	−4.60 (14)
S2—Co—N2—C11	−177.39 (18)	C21—N3—C22—C23	110.7 (4)
S3—Co—N2—C11	69.8 (4)	C24—N3—C22—C23	−65.5 (4)
S4—Co—N2—C11	88.22 (18)	C21—N3—C24—C25	124.4 (3)
S1—Co—N2—C11	−100.38 (18)	C22—N3—C24—C25	−59.4 (4)
C12—N1—C1—C2	1.5 (4)	C32—N4—C31—S3	1.8 (4)
Co—N1—C1—C2	−176.9 (2)	C34—N4—C31—S3	−179.5 (3)
N1—C1—C2—C3	0.5 (5)	C32—N4—C31—S4	−178.0 (2)
C1—C2—C3—C4	−1.1 (5)	C34—N4—C31—S4	0.7 (4)
C2—C3—C4—C12	−0.4 (4)	Co—S3—C31—N4	−176.0 (3)
C2—C3—C4—C5	179.2 (3)	Co—S3—C31—S4	3.78 (13)
C3—C4—C5—C6	179.5 (3)	Co—S4—C31—N4	176.0 (3)
C12—C4—C5—C6	−1.0 (4)	Co—S4—C31—S3	−3.77 (13)
C4—C5—C6—C7	−0.4 (5)	C31—N4—C32—C33	93.6 (4)
C5—C6—C7—C11	0.9 (4)	C34—N4—C32—C33	−85.1 (4)
C5—C6—C7—C8	179.4 (3)	C31—N4—C34—C35	89.0 (4)
C11—C7—C8—C9	0.5 (4)	C32—N4—C34—C35	−92.2 (4)

### Table 2 C-H···F contacts in (I) (Å, deg)

**Table:**

D-H	A	D-H	H···A	D···A	D-H..A
C10-H10A	F1^i^	0.94	2.31	3.169 (4)	151.2
C2-H2A	F2^ii^	0.94	2.43	3.281 (4)	150.9
C6-H6A	F4^iv^	0.94	2.44	3.053 (4)	122.9

Symmetry codes (i) x,y,-1+z (ii)  -1/2+x,1/2-y,-1/2+z (iii)  1-x,1-y,1-z (iv)  1/2-x,-1/2+y,1/2-z
